# Robotic Pancreaticoduodenectomy for Pancreatic Head Tumour: A Single-Centre Analysis

**DOI:** 10.3390/cancers16244243

**Published:** 2024-12-20

**Authors:** Vera Hartman, Bart Bracke, Thiery Chapelle, Bart Hendrikx, Ellen Liekens, Geert Roeyen

**Affiliations:** 1 Department of Hepatopancreaticobiliary, Endocrine and Transplantation Surgery, Antwerp University Hospital, 2650 Edegem, Belgium; 2 Faculty of Medicine and Health Sciences, University of Antwerp, 2650 Edegem, Belgium

**Keywords:** pancreaticoduodenectomy, robotic, minimally invasive

## Abstract

**Background**: The robotic approach is an appealing way to perform minimally invasive pancreaticoduodenectomy. We compare robotic cases’ short-term and oncological outcomes to a historical cohort of open cases. **Methods**: Data were collected in a prospective database between 2016 and 2024; complications were graded using the ISGPS definition for the specific pancreas-related complications and the Clavien–Dindo classification for overall complications. Furthermore, the Comprehensive Complication Index was calculated. All patients undergoing pancreaticoduodenectomy were included, except those with acute or chronic pancreatitis, vascular tumour involvement or multi-visceral resections. Only the subset of patients with malignancy was regarded for the oncologic outcome. **Results**: In total, 100 robotic and 102 open pancreaticoduodenectomy cases are included. Equal proportions of patients have a main pancreatic duct ≤3 mm (*p* = 1.00) and soft consistency of the pancreatic remnant (*p* = 0.78). Surgical time is longer for robotic pancreaticoduodenectomy (*p* < 0.01), and more patients have delayed gastric emptying (44% and 28.4%, *p* = 0.03). In the robotic group, the number of patients without any postoperative complications is higher (*p* = 0.02), and there is less chyle leak (*p* < 0.01). Ninety-day mortality, postoperative pancreatic fistula, and postpancreatectomy haemorrhage are similar. The lymph node retrieval and R0 resection rates are comparable. **Conclusions**: In conclusion, after robotic pancreaticoduodenectomy, remembering all cases during the learning curve are included, less chyle leak is observed, the proportion of patients without any complication is significantly larger, the surgical duration is longer, and more patients have delayed gastric emptying. Oncological results, i.e., lymph node yield and R0 resection rate, are comparable to open pancreaticoduodenectomy.

## Simple Summary

Robotic pancreaticoduodenectomy has gained more widespread interest over the past decade. Although the robotic platform’s three-dimensional stereoscopic view and dexterity in its endo-wristed instruments present an appealing minimally invasive approach for pancreaticoduodenectomy, data on the short-term postoperative outcomes and oncological safety are scarce. To examine these outcomes and the oncological results, a comparison is made between the robotic pancreaticoduodenectomy procedures performed since the adaptation of the robotic platform at Antwerp University Hospital and the open pancreaticoduodenectomy cases performed before. This comparison reveals an equal 90-day mortality rate, an equal number of postpancreatectomy haemorrhage, pancreatic fistula, and severe complications; more patients without any complications; and less chyle leak in the robotic group and a higher number of patients with delayed gastric emptying in the robotic cohort. Furthermore, oncological outcome parameters, such as lymph node retrieval and R0 resection rate, are comparable.

## 1. Introduction

Minimally invasive pancreaticoduodenectomy was first introduced in the late nineties after the successful implementation of laparoscopy in many other surgical procedures. Nevertheless, laparoscopic pancreaticoduodenectomy today remains a challenging technique and requires a long learning curve to master. Only limited evidence supports the use of laparoscopy for pancreaticoduodenectomy, with several authors demonstrating a faster recovery when comparing laparoscopy to an open approach [1,2]. The LEOPARD-2 trial, a Dutch RCT comparing open and laparoscopic pancreaticoduodenectomy for pancreatic head or periampullary tumours, was prematurely terminated by the safety monitoring board due to an alarming 90-day mortality rate of 10% in the laparoscopic group, compared to 2% in the open group [3]. Furthermore, the oncologic safety of laparoscopic pancreaticoduodenectomy remains unclear.

In 2001, Giulianotti performed the first robotic pancreaticoduodenectomy (RPD) [4]. RPD is considered the natural evolution of the laparoscopic approach, with its enhanced three-dimensional, stereoscopic, and stable view; its dexterity in the endo-wristed instruments; and its improved ergonomics. This technique has slowly gained interest and has been more widely adopted recently. Several single-centre, non-randomised studies show that RPD might have comparable or even better surgical and oncological outcomes than open and laparoscopy [5,6,7,8,9,10,11]. The first RCT comparing robotic and open pancreaticoduodenectomy (OPD), the EUROPA trial, published in The Lancet Regional Health in 2024, shows that robotic and open pancreaticoduodenectomy are safe procedures [12]. However, further RCT trials are required to uncover potential advantages of the robotic approach in terms of perioperative and long-term outcomes and to justify the high costs of the robotic platform and instruments [13].

RPD was introduced to Antwerp University Hospital in August 2020. Following an intensive training programme comprised of online video material, attending the 2019 Hands-on Course organised by the European Consortium for Minimally Invasive Pancreatic Surgery in Amsterdam, a large number of virtual training hours on the Intuitive XI platform, workshops on both cadaveric and pig models at the Orsi Academy in Ghent, and a case observation with Prof. M. Besselink at Amsterdam UMC. Consequently, approximately 65% of all pancreatic surgery in our department today is performed robotically.

This paper analyses the results of the initial RPD cases performed between August 2020 and January 2024. These cases are compared to OPD cases performed before introducing the robotic approach (i.e., before August 2020) to avoid selection bias. Both the perioperative outcome and the oncological results are examined.

## 2. Materials and Methods

Since 2016, data on all pancreatic resections have been collected in a prospective database. Postoperative complications are graded using the ISGPS definitions for the specific pancreas-related complications, and the Clavien–Dindo classification is used for postoperative complications in general [14,15,16,17,18]. Furthermore, the Comprehensive Complication Index (CCI) is calculated based on this [19]. All patients undergoing pancreaticoduodenectomy are included: open cases between January 2016 and August 2020 and robotic cases between August 2020 and January 2024. Patients with underlying acute or chronic pancreatitis, vascular involvement of the tumour or multi-visceral resection have been excluded. Only the subset of patients with pancreatic or periampullary cancer is regarded for the oncological outcome. The surgical duration is measured from the incision to the final closing stitch. Perioperative blood loss is estimated based on the amount in the suction device and the use of surgical pads. To assess the risk for complications, the age-adjusted Charlson Comorbidity Score [20] is calculated, and, during surgery, the consistency of the pancreatic remnant is assessed (scored as either “soft” or “hard”), and the diameter of the main pancreatic duct is measured. The pancreatic duct diameter is measured in vivo. In open pancreaticoduodenectomy, various probe sizes are used. In the robotic group, the diameter can be measured by comparing it to the length of the robotic needle driver and by using three different sizes of silicone stents placed in the main pancreatic duct.

The surgical technique has been standardised in both the open and robotic approach. For OPD, the procedure is performed by two surgeons. A transverse incision is made; next, using a sealing device and bipolar scissors, the lesser sac is opened by dissecting the gastrocolic ligament, and the superior mesenteric vein is exposed at the inferior border of the pancreatic neck. Then, a Kocher manoeuvre is performed to expose the inferior vena cava, after which the duodenum and pancreatic head are further mobilised. Next, a cholecystectomy is performed, and the porta hepatis is dissected: the right gastric and gastroduodenal artery are divided after lymph node dissection, the bile duct is divided, and the portal vein is exposed. Then, the stomach is divided using a tristaple technology stapler, just proximal to the pylorus. After that, the pancreatic neck is divided using cautery. The proximal jejunum is divided, and the uncinate process is dissected while visualising the superior mesenteric artery. In the uncinate process, an artery-first approach is used in patients with a larger tumour. Reconstruction of the pancreas is performed with a “dunking” technique using interrupted absorbable monofilament 4/0 sutures after placing a silicone stent in the main pancreatic duct. The hepaticojejunostomy is performed using continuous or interrupted absorbable monofilament 5/0 or 6/0 sutures, depending on the diameter of the bile duct. Lastly, the gastroenterostomy is made end-to-side 40 cm distal to the hepaticojejunostomy, using two layers of continuous absorbable monofilament 4/0 sutures.

For RPD, the Intuitive XI platform^®^ (Sunnyvale, CA, USA) is used. The patient is placed in a supine position with the legs spread, with 15° antitrendelenburg and 5° left tilt. The procedure is always performed by two dedicated surgeons: the first performing the dissection phase at the robot console and the second performing the reconstruction phase. A Veress needle is placed at Palmer’s point, and a pneumoperitoneum of 15 mmHg is maintained during port placement and lowered to 12 mmHg for the remainder of the procedure. Four 8 mm robot ports are placed, 8 cm apart, on a horizontal line at or above the umbilicus (depending on the patient’s posture), and, additionally, a 12 mm assistant port is placed below the umbilicus, and a 5 mm port is placed 8 cm to the right. First, the falciform ligament is sutured to the abdominal wall to expose the liver hilum. A cautery hook and robotic sealing device (Vessel Sealer^®^, Sunnyvale, CA, USA) are utilised to dissect. Next, the duodenum is mobilised from the ligament of Treitz, and 80 cm of jejunum is measured and fixed to the abdominal wall at the left side, marking the biliary side, enabling easy identification of the jejunal limb for the gastroenterostomy after the pancreatic head resection is completed. The following dissection steps are the same as for the open approach: opening of the lesser sac and exposure of the superior mesenteric vein; Kocher manoeuvre and mobilisation of the duodenum and pancreatic head; dissection of the hepatic hilum with division of the gastroduodenal artery and bile duct; transection of the stomach, the pancreatic neck, and the jejunum; and finally, the dissection of the uncinate process. The specimen is then extracted through an enlarged incision at the 12 mm assistant port below the umbilicus, and the port is replaced using an Alexis^®^ wound protector. After the surgeons have switched positions, the reconstruction of the pancreas is performed with a “dunking” end-to-side layer of interrupted absorbable braided 3/0 sutures, leaving a silicone stent in the main pancreatic duct. The hepaticojejunostomy is performed using either continuous barbed 4/0 sutures or interrupted absorbable monofilament 5/0 or 6/0 sutures, depending on the diameter of the bile duct. Finally, the gastroenterostomy is performed end to side using a single layer of continuous absorbable barbed 3/0 sutures.

Data are analysed using SPSS v27. The data are expressed as medians [range] for non-normally distributed continuous variables, whereas categorical data are expressed as numbers (%). Meanwhile, the Mann–Whitney U test for non-normally distributed variables is used to compare medians for outcome parameters, while Fisher’s exact test is used to compare categorical data. A *p*-value < 0.05 is considered statistically significant.

## 3. Results

### 3.1. Patient Characteristics

In total, 100 patients are included in the RPD group, all having undergone their surgery after August 2020, when the robot was first used for pancreaticoduodenectomy cases. In other words, the cases during the learning curve are also included in this cohort. In the open group, 102 patients are included. All open cases were performed before the introduction of the robotic approach. Table 1 summarises patient characteristics. This shows two significant differences between both patient groups: firstly, the robotic population is older, and secondly, more patients with preoperative biliary drainage, either endoscopically or with percutaneous drainage, are included in the robotic group. On the other hand, the age-adjusted Charlson Comorbidity Score does not differ between groups, and the proportion of patients with a soft pancreatic remnant is similar. Additionally, the median diameter of the main pancreatic duct is 4 [1–10] mm in the robotic group and 4 [1–12] mm in the open group (*p* = 0.32). The different indications for surgery are equally divided over both approaches.

Only patients with an underlying pancreatic or periampullary tumour (i.e., pancreatic adenocarcinoma, cholangiocarcinoma, ampullary or duodenal carcinoma, and pancreatic neuroendocrine tumours (pNET)) are regarded for the oncological outcome. Eighty-two patients were analysed in the robotic group, and 78 were in the open cohort. The patient characteristics for this oncological subset are listed in Table 2. Here, no significant differences are observed between the robotic and open groups.

### 3.2. Short-Term Postoperative Outcomes

**Figure 1:**
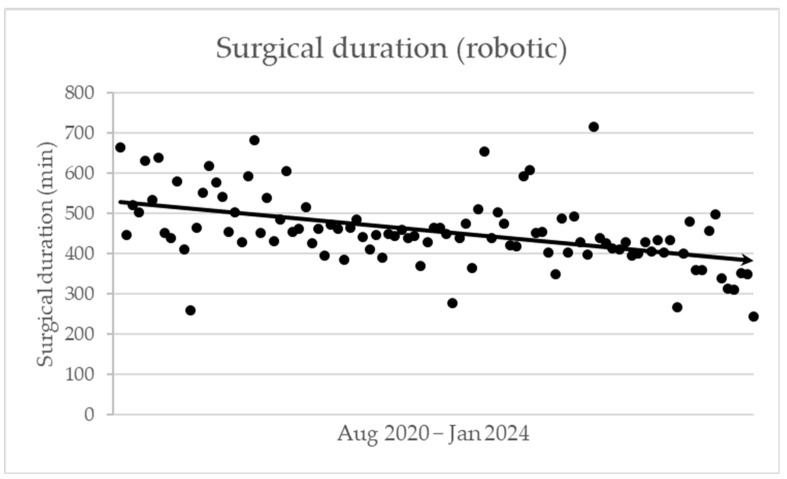
Evolution of surgical duration of robotic procedures.

Table 3 summarises the peri- and postoperative results. Considering the perioperative results, the robotic approach’s surgical duration is significantly longer. Since the robotic cases include all procedures during the learning curve, we add Figure 1, which shows a declining trend in the procedure length over time. Furthermore, the conversion rate in the robotic group is 16%, and the estimated blood loss is equal for both groups. To summarise the reasons for conversion in the RPD group: In five patients, severe pancreatitis due to preoperative biliary stenting or biopsy required early conversion to open surgery. Only three patients required urgent conversion due to bleeding during the dissection of the uncinate process from the portomesenteric vein. Another two patients faced difficulty with hepatic artery dissection due to prior neoadjuvant treatment. Four patients were converted during the reconstruction phase due to a very soft and lipomatic pancreas. Finally, two patients had tumours extending into the mesocolon, making safe dissection challenging.

**Figure 2:**
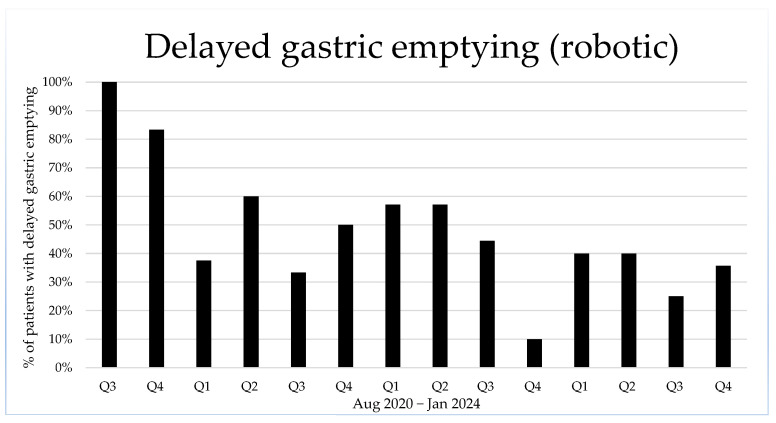
Percentage of delayed gastric emptying in robotic procedures, shown per quartile.

Next, we consider the complications specific to pancreatic surgery. Postpancreatectomy haemorrhage (PPH) and clinically significant (grade B or C) postoperative pancreatic fistula rates (CR-POPF) are comparable in both robotic and open surgery. Chyle leaks are more frequent after OPD (*p* < 0.01), which is most evident for grade B chyle leaks. On the other hand, more delayed gastric emptying is observed after RPD, particularly more severe grade C delayed gastric emptying. Figure 2 shows the positive evolution in RPD cases complicated by delayed gastric emptying during the study period.

When we focus on all types of postoperative complications, the number of grade 3 or above Clavien–Dindo complications is similar after both approaches. In contrast, there are significantly more patients without complications (Clavien–Dindo 0) after the robotic approach: 19%, compared to 8% after open surgery (*p* = 0.02). The median CCI is 22.6 [0–100] in the robotic group, which is lower than 29.6 in the open group, but this difference does not reach significance. Ninety-day mortality, the total length of hospital stay, and the length of stay in the ICU are comparable for both approaches.

### 3.3. Oncological Outcomes

Oncological parameters are shown in Table 4. The median number of resected lymph nodes is 19 for both surgical approaches (*p* = 0.87). R0 resection margins are achieved in 89% with both techniques (*p* = 1.00).

## 4. Discussion

This is a retrospective analysis of prospectively collected data, with a risk of selection bias. Nevertheless, we believe we can avoid this issue by using strict inclusion criteria. In the open surgery group, all patients had their pancreaticoduodenectomy before August 2020, before the robotic approach was introduced, and we included cases that would have been performed robotically had it been available. Patients with underlying acute or chronic pancreatitis, vascular involvement of the tumour, or multi-visceral resection were excluded. Considering the results in Table 1 and Table 2, risk factors for surgical complications are comparable. For example, the age-adjusted Charlson Comorbidity Score and BMI are similar for both approaches. Furthermore, there is no difference between the proportion of patients with a “soft” consistency of the pancreatic remnant or a normal main pancreatic duct (≤3 mm). These have been described as important risk factors for developing CR-POPF [21,22]. However, two significant differences are observed. Firstly, the robotic population is older than the open group, 71 [30–89] compared to 66.5 [28–88] years. Secondly, more patients had undergone biliary drainage preoperatively; perhaps this can be explained by the COVID-19 pandemic, causing a shortage of hospital staff, particularly OR nurses, which has lengthened the time between diagnosis and surgery, necessitating more frequent preoperative biliary drainage.

The high incidence of delayed gastric emptying after RPD (44%) is a matter of concern. Surgical procedure has been standardised in both the open and the robotic approach, with only slight adaptations of the anastomosis technique for pancreatojejunostomy and hepaticojejunostomy: the pancreatojejunostomy is made end to side with a “dunking” technique using interrupted sutures 3/0 or 4/0, monofilament for open, and braided for robotic surgery; the robotic hepaticojejunostomy is performed identically to the open approach, except for very dilated bile ducts, where a 4/0 continuous barbed suture is preferred. In contrast, the robotic gastroenterostomy has changed considerably throughout the study period. In the first six months, a continuous double-layer end-to-side anastomosis with 4/0 monofilament sutures was performed through a subcostal incision in the right upper quadrant. Because of delayed gastric emptying, this technique was altered to a full robotic anastomosis using a linear endoscopic stapler and a continuous monofilament suture to close the defect. Approximately one year later, after two bleeding episodes from the stapled anastomosis and an ongoing high incidence of delayed gastric emptying, the gastroenterostomy technique was finally adjusted to an end-to-side anastomosis, removing the stapled gastric margin, using a monolayer, continuous 3/0 barbed suture. With these adjustments in the anastomosis technique, delayed gastric emptying has declined, as shown in Figure 2. Previous studies described a lower or comparable incidence of delayed gastric emptying [6,23,24]. However, the EUROPA trial reports a higher incidence of delayed gastric emptying (34.4% after RPD, compared to 6% after OPD, *p* < 0.05) [12].

Shyr et al. reported a higher incidence of chyle leak after RPD than open [9,25]. In contradiction, in this study, RPD causes less chyle leak. The first possible explanation is the improved vision with the robotic system, with its enhanced, three-dimensional, stereoscopic, 10x enlarged view that is not dependent on the stamina of the surgical assistant, ensuring better visualisation and more careful manipulation of lymphatic vessels. The second possibility is that the sealing device used in this study, Vessel Sealer Intuitive^®^ (Sunnyvale, CA, USA), may have a better sealing strength than the device used in the open approach (Ligasure Dolphin tip, Medtronic^®^ Minneapolis, MN, USA).

In agreement with several other reports [6,24,26,27,28], this study shows a similar incidence of PPH, CR-POPF, and 90-day mortality after RPD compared to OPD. Cai et al. suggest that, although the numbers were comparable, CR-POPF after RPD had less clinical impact than OPD [29]. The EUROPA trial also revealed a comparable CCI, CR-POPF, and PPH [12].

This study shows no significant difference in the length of hospital stay between the robotic and open approaches, although faster patient mobilisation is possible. The primary factor that influences the length of stay in these patients is the time to adequate oral intake, which is a factor that has not changed with the introduction of the robot in our centre. We believe that further, more rigorous application of ERAS principles and the recent changes in the gastroenterostomy technique will improve these results.

Our data suggest oncological safety regarding perioperative results: both R0 resection rate and lymph node retrieval are equal for both approaches. A meta-analysis by Podda et al., comparing 1593 RPD and 12046 OPD in patients with benign and malignant periampullary disease, showed equivalent results in terms of retrieved lymph nodes (19 ± 10 for RPD vs. 17 ± 10 for OPD, *p* = 0.22) [30]. Chen et al. reported a propensity score-matched analysis of 103 RPD and 206 OPD cases for pancreatic adenocarcinoma, showing a longer median disease-free survival following RPD (18.5 months) compared to 14.0 months after OPD (*p* = 0.01). RPD was independently associated with improved overall survival (HR 0.7, 95%CI 0.52–0.94, *p* = 0.02) and disease-free survival (HR0.66, 95%CI 0.50–0.88, *p* = 0.01) [31]. Randomised controlled trials in selected patient populations are needed. The DIPLOMA-2 study will soon be published; this international, multicentre, patient-blinded RCT, initiated by the European Consortium on Minimally Invasive Pancreatic Surgery (E-MIPS), compares minimally invasive and OPD in patients with pancreatic and periampullary neoplasm [32]. A continuation of this study is the DIPLOMA-2x2 study, which will specifically compare RPD to OPD for primary resectable pancreatic head cancer, and the primary outcome parameter is the radicality of the resection with blinding of the pathologist.

## 5. Conclusions

We must be careful about drawing conclusions from these results and remember that all cases during the learning curve are included in the robotic group, whereas the learning curve was already surpassed in the open group. In the robotic group, surgical duration is longer, even though a clear declining trend is observed. More patients experience delayed gastric emptying after robotic surgery. On the other hand, less chyle leak is seen after RPD, and the proportion of patients with no complication is significantly larger than after open surgery. Incidence of PPH and CR-POPF, length of ICU and hospital stay, and 90-day mortality are comparable. The number of resected lymph nodes and the R0 resection rate are equal.

Therefore, we dare to conclude that regarding short-term postoperative outcomes, RPD is a safe procedure, even during the learning curve, with an equal to or better outcome than the open approach. Furthermore, perioperative oncological outcome parameters are equivalent to OPD.

## Figures and Tables

**Table 1:** Patient characteristics (malignant and premalignant indications).

	RPD (n = 100)	OPD (n = 102)	*p*-Value
Age (years)	71 [30–89] ^1^	66 [28–88]	0.03
Sex			
Male	48 (48.0)	54 (52.9)	0.57
Female	52 (52.0)	48 (47.1)	
BMI (kg/m^2^)	25 [17–37]	25 [15–39]	0.47
Age-adjusted CCS ^2^	3 [0–8]	3 [0–11]	0.31
Biliary drainage	51 (51.0)	30 (29.4)	<0.01
Parenchymal consistency			
“soft”	52 (52.0)	56 (54.9)	0.78
“hard”	48 (48.0)	46 (45.1)	
Main pancreatic duct			
≤3 mm	37 (37.0)	38 (37.3)	1.00
>3 mm	63 (61.8)	64 (64.0)	
Pathology			
Pancreatic adenocarcinoma	44 (44.0)	50 (49.0)	0.48
Distal cholangiocarcinoma	13 (13.0)	7 (6.9)	0.16
pNET	8 (8.0)	7 (6.9)	0.79
Other malignancies ^3^	13 (13.0)	18 (17.7)	0.44
Premalignant lesion ^4^	22 (22.0)	20 (19.6)	0.73

^1^ Categorical data expressed as numbers (percentage)–continuous data expressed as median [range]; ^2^ Comprehensive Complication Index; ^3^. Ampullary carcinoma, duodenal carcinoma, GIST; ^4^ IPMN, mucinous cystadenoma.

**Table 2:** Patient characteristics (malignant indications only).

	RPD (n = 82)	OPD (n = 78)	*p*-Value
Age (years)	71.5 [30–89] ^1^	68.5 [28–88]	0.23
Sex			
Male	36 (46.2)	42 (51.2)	0.53
Female	42 (53.8)	40 (48.8)	
BMI (kg/m^2^)	25 [17–39]	25 [15–39]	0.93
Age-adjusted CCS ^2^	4 [0–11]	3 [0–8]	0.46
Biliary drainage	48 (61.5)	29 (35.4)	<0.01
Parenchymal consistency			
“soft”	41 (52.6)	43 (52.4)	1.00
“hard”	37 (47.4)	39 (47.6)	
Main pancreatic duct			
≤3 mm	34 (43.6)	28 (34.1)	0.32
>3 mm	44 (56.4)	54 (65.9)	
Neoadjuvant treatment	6 (7.7)	4 (4.8)	0.53
CA19.9 (kE/L)	46.2 [0–8446.0]	86.3 [1.9–2597.4]	0.16

^1^ Categorical data expressed as numbers (percentage)—continuous data expressed as median [range]; ^2^ Comprehensive Complication Index.

**Table 3:** Peri- and postoperative outcomes (malignant and premalignant indications).

	RPD (n = 100)	OPD (n = 102)	*p*-Value
Surgical duration (min)	446 [244–716] ^1^	268 [162–528]	<0.01
Blood loss (mL)	300 [50–2000]	300 [50–2000]	0.34
Conversion to open	16 (16.0)	NA	
PPH ^2^	13 (13.0)	19 (19.6)	0.34
Grade A	2 (2.0)	5 (4.9)	0.44
Grade B	9 (9.0)	6 (5.9)	0.43
Grade C	1 (1.0)	7 (6.9)	0.06
POPF ^3^	8 (8.0)	11 (10.8)	0.63
Grade B	2 (2.0)	6 (5.9)	0.28
Grade C	6 (6.0)	5 (4.9)	0.77
Chyle leak	15 (15.0)	37 (36.3)	<0.01
Grade A	14 (14.0)	25 (24.5)	0.06
Grade B	1 (1.0)	9 (8.9)	0.02
Grade C	0 (0.0)	2 (2.0)	0.50
Delayed gastric emptying	44 (44.0)	29 (28.4)	0.03
Grade A	15 (15.0)	12 (11.8)	0.54
Grade B	14 (14.0)	14 (13.7)	1.00
Grade C	15 (15.0)	5 (4.9)	0.02
Clavien–Dindo ≥ 3	39 (39.0)	35 (34.3)	0.46
Clavien–Dindo = 0	19 (19.0)	8 (7.8)	0.02
CCI ^4^	22.6 [0–100]	29.6 [0–100]	0.07
90-day mortality	5 (5.0)	5 (4.9)	1.00
Length of ICU stay	2 [1–65]	2 [1–40]	0.03
Total length of stay	17 [8–155]	17 [8–73]	0.62

^1^ Categorical data expressed as numbers (percentage)—continuous data expressed as median [range]; ^2^ postpancreatectomy haemorrhage; ^3^ postoperative pancreatic fistula; ^4^ comprehensive Complication Index.

**Table 4:** Oncological outcomes (malignant indications only).

	RPD (n = 82)	OPD (n = 78)	*p*-Value
No. lymph nodes	19 [6–48] ^1^	19 [6–49]	0.87
Resection margins			
R0	70 (89.7)	73 (89.0)	1.00
R1	8 (10.3)	9 (11.0)	

^1^ Categorical data expressed as numbers (percentage)—continuous data expressed as median [range].

## Data Availability

All data are available on request.
